# Predicting *Salmonella* MIC and Deciphering Genomic Determinants of Antibiotic Resistance and Susceptibility

**DOI:** 10.3390/microorganisms12010134

**Published:** 2024-01-10

**Authors:** Moses B. Ayoola, Athish Ram Das, B. Santhana Krishnan, David R. Smith, Bindu Nanduri, Mahalingam Ramkumar

**Affiliations:** 1 Department of Comparative Biomedical Sciences, College of Veterinary Medicine, Mississippi State University, Starkville, MS 39762, USA; mba185@msstate.edu (M.B.A.); ar2903@msstate.edu (A.R.D.); sb3700@msstate.edu (B.S.K.); bnanduri@cvm.msstate.edu (B.N.); 2 Department of Population Medicine, College of Veterinary Medicine, Mississippi State University, Starkville, MS 39762, USA; david.smith@msstate.edu; 3 Department of Computer Science and Engineering, Mississippi State University, Starkville, MS 39762, USA

**Keywords:** *Salmonella*, antibiotics, MIC, machine learning

## Abstract

*Salmonella* spp., a leading cause of foodborne illness, is a formidable global menace due to escalating antimicrobial resistance (AMR). The evaluation of minimum inhibitory concentration (MIC) for antimicrobials is critical for characterizing AMR. The current whole genome sequencing (WGS)-based approaches for predicting MIC are hindered by both computational and feature identification constraints. We propose an innovative methodology called the “Genome Feature Extractor Pipeline” that integrates traditional machine learning (random forest, RF) with deep learning models (multilayer perceptron (MLP) and DeepLift) for WGS-based MIC prediction. We used a dataset from the National Antimicrobial Resistance Monitoring System (NARMS), comprising 4500 assembled genomes of nontyphoidal *Salmonella*, each annotated with MIC metadata for 15 antibiotics. Our pipeline involves the batch downloading of annotated genomes, the determination of feature importance using RF, Gini-index-based selection of crucial 10-mers, and their expansion to 20-mers. This is followed by an MLP network, with four hidden layers of 1024 neurons each, to predict MIC values. Using DeepLift, key 20-mers and associated genes influencing MIC are identified. The 10 most significant 20-mers for each antibiotic are listed, showcasing our ability to discern genomic features affecting *Salmonella* MIC prediction with enhanced precision. The methodology replaces binary indicators with k-mer counts, offering a more nuanced analysis. The combination of RF and MLP addresses the limitations of the existing WGS approach, providing a robust and efficient method for predicting MIC values in *Salmonella* that could potentially be applied to other pathogens.

## 1. Introduction

*Salmonella* spp., one of the leading causes of foodborne illness all around the world, can contaminate a wide range of food products including meat, poultry, eggs, dairy, fruits, and vegetables. When consumed, contaminated food can cause salmonellosis and gastroenteritis with symptoms such as nausea, diarrhea, abdominal pain, and fever. According to the Centers for Disease Control and Prevention (CDC), *Salmonella* is responsible for an alarming global toll, causing an estimated 150 million cases of illness and resulting in 60,000 fatalities annually [1]. In the United States alone, salmonellosis accounts for more than one million illnesses and approximately 420 deaths each year [2]. While many cases of foodborne illnesses may naturally resolve or, in severe instances, can be treated with antibiotics, the emergence of AMR poses a significant challenge to effective therapeutic strategies.

Due to the concerted efforts of the National Antimicrobial Resistance Monitoring System (NARMS, [3]), a collaborative effort between the United States Department of Agriculture (USDA), CDC, and Food and Drug Administration (FDA) since 2002, comprehensive surveillance has been in place. Surveillance focuses on tracking the prevalence of pathogens responsible for foodborne illnesses, including *Salmonella*, *Campylobacter*, *Escherichia*, and *Enterococcus*. NARMS assesses foodborne pathogen susceptibility to 40 antibiotics (15 for both *Salmonella* and *Escherichia*, 9 for *Campylobacter*, and 16 for *Enterococcus*), resulting in a wealth of MIC information for these pathogens. The available MIC data are critical for effectively inhibiting the growth of these pathogens. In addition to determining the MICs, NARMS has also been actively expanding its data collection efforts by gathering WGS from randomly selected isolates of foodborne pathogens.

The NARMS program has achieved considerable success by delivering timely insights into the trends of antibiotic resistance [4,5]. However, there is a concerning trend showing that AMR in *Salmonella* has shown a steady increase since 2015, especially in poultry chickens (ceca during slaughtering, chicken carcass/parts during processing and inspection, and even sampled retail chickens that have been sold to the public) [3]. The reports have gone a step further in pinpointing recent resistance trends, particularly regarding ciprofloxacin, which is among the first-line antibiotics for treating *Salmonella* infections [6,7,8], and some other antibiotics such as chloramphenicol, trimethoprim-sulfamethoxazole, sulfisoxazole, nalidixic acid, streptomycin, and tetracycline. Determining the MIC quickly with minimal lab testing, while making accommodations for the genetic diversity within pathogenic strains, is essential to ensure that treatments can be customized, timely, and effective.

MIC values for bacterial strain and antibiotic pairs are traditionally determined using agar or broth dilution methods, described by the Clinical and Laboratory Standards Institute [9,10,11,12]. However, traditional methods for determining the MIC of antimicrobial agents are hindered by time-consuming processes, posing challenges in promptly addressing serious infections [10] as they often involve substantial hands-on labor, involving tasks like plate preparation and serial dilutions, increasing the risk of errors and operator-dependent variability. Publicly available WGS data, paired with clinical AMR metadata, has enabled the use of machine learning (ML) to predict MIC values and track temporal trends, eliminating sole reliance on AMR databases. Using short nucleotide sequences (referred to as k-mers or genomic features interchangeably henceforth, where k denotes the sequence length) as features and laboratory-derived MIC values as labels, precise predictions of susceptibility or resistance to antibiotics are made, even without prior genetic information about the organisms [13,14,15,16,17]. The WGS data from NARMS has been used to predict the MIC values of 15 commonly monitored antibiotics for *Salmonella* using XGBoost [18,19], with an average accuracy of 95% within a ±1 2-fold dilution step of the laboratory-determined values. However, the study identified k-mers that play a crucial role in MIC prediction only by using a subset of the samples owing to computational limitations. A random forest and a neural network model in parallel were used to predict susceptibility/resistance in *Mycobacterium tuberculosis*, *Escherichia coli*, *Salmonella enterica*, and *Staphylococcus aureus* [20], while Adaboost was used to predict resistance to carbapenem, methicillin, and beta-lactam in *Acinetobacter baumannii*, *Staphylococcus aureus*, and *Streptococcus pneumoniae*, respectively [21], using data from the PATRIC database [22]. Similarly, random forest, support vector machine, and XGBoost were used to predict cefoxitin resistance in S. aureus [23] and logistic regression was used to predict resistance to ethambutol, ethionamide, isoniazid, kanamycin, ofloxacin, rifampicin, and streptomycin in *M. tuberculolis* and *S. aureus* [24]. The existing approach, which employs WGS to predict MIC, has made significant strides. However, it still faces some limitations. One of these limitations is the substantial computational memory required for processing WGS data and making MIC predictions. For example, Nguyen et al. successfully predicted MIC using 10-mers and analyzed 4500 genomes. However, when attempting to identify crucial k-mers through BLAST searches, their scope was limited to 15-mers and 1000 genomes [19]. Furthermore, the current approach faces challenges in distinguishing whether the identified k-mers are associated with low or high MIC values. These challenges highlight the need for more efficient and precise methods to address these shortcomings and enhance our understanding of MIC prediction.

In this study, we have developed the “Genome Feature Extractor Pipeline” to address challenges associated with using 10-mers for MIC prediction. Our pipeline transforms approximately one million possible 10-mers into a more manageable set of a few tens of thousands 20-mers, effectively capturing genomic regions influencing MIC values. It distinguishes the contributions of essential 20-mers and genes, crucial for understanding susceptibility and resistance in a dataset of 4500 genomes. Many of the predictive k-mers and genes align with known resistance mechanisms. Additionally, our model reveals potential antibiotic resistance-related genes, although these require validation through experiments.

## 2. Materials and Methods

### 2.1. Data Curation and Analysis Pipeline

**Figure 1:**
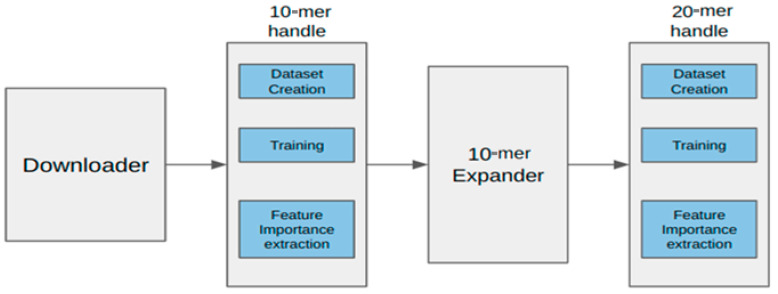
The four step genome feature extractor pipeline. (1) Downloader: batch-downloads the annotated genome dataset. (2) The 10-mer handle: creates 10-mer count vectors from each sample based on a model that chooses a subset of important 10-mers. (3) The 10-mer expander: generates 20-mers from 10-mers. (4) The 20-mer handle: creates the dataset and performs training and extraction of important 20-mers from the extended dataset.

The NARMS dataset used in this study has 4500 assembled and annotated genomes (used to identify features) of nontyphoidal *Salmonella* along with their associated MIC metadata information (labels). We identified genomic features that are most predictive of MIC for the 15 antibiotics, listed in Table 1, for *Salmonella* [3]. We used the frequencies (number of occurrences) of a specific subset of 20-mer to predict MICs. With 4^20^ (≈10^12^) possible 20-mers, and a dataset of *Salmonella* genomes with an average of 5 × 10^6^ base pairs, the search to obtain occurrence frequencies or counts of the k-mers is almost impossible. Thus, we identified the subset of k-mers using the 4-step process, in the genome feature extraction pipeline depicted in Figure 1. The first step was to batch-download the annotated genomes from the Bacterial and Viral Bioinformatics Resource Center [22]. In the second step, we chose the set of “important” 10-mers. The total number of possible 10-mers is only of the order of a million (4^10^ = (2^10^) ≈ (10^3^)^2^), and for each sample genome sequence, we extracted the 10-mer counts, creating a count data of size 4500 × 10^6^. Counts of k-mer occurrences, though easy to calculate, have shown promise in MIC predictions [25], although other features such as the individual or joint (co-occurrence) positional behavior of k-mers are slightly more computationally intensive, and may provide further biological insights.

**Figure 2:**
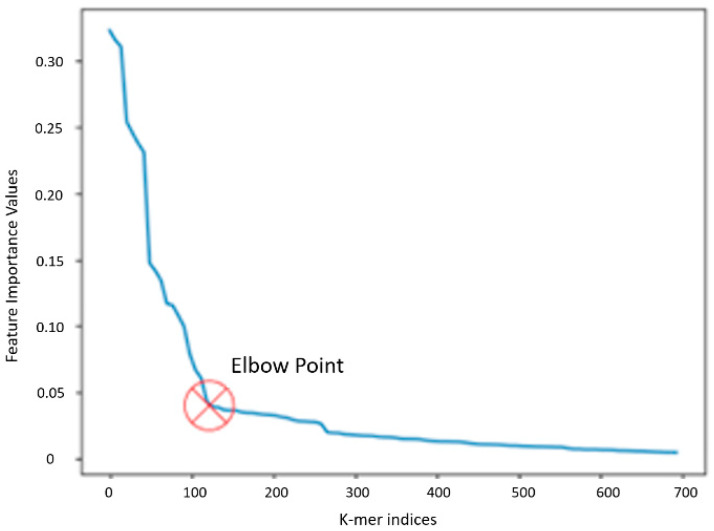
Filtering 10-mers based on feature importance plot and the elbow point, shown here for Ampicillin.

The RF (additional details in Section 2.2) is a collection of decision trees that learn both individual and joint feature interactions and is nonparametric and computationally efficient. This prediction problem fits the limited sample sizes (4500) and high-dimensional (10^6^ for 10-mers) feature space case, where the RF is a better choice than a deeper neural network. Feature selection or dimensionality reduction approaches explicitly calculate a subset of input features that best describe (or estimate) the target variable (MIC). We compute each feature’s contribution, called “feature importance”, based on the Gini index [26]. Figure 2 shows an example plot of the feature importance values of 10-mers for the antibiotic ‘AMP’. An inflection point or “elbow point” is the point at which we observe the shift in the gradient of the importance values from a large negative value to smaller ones, indicating the saturation of the representation. We chose the 10-mers corresponding to the indices with importance values above the elbow points, as depicted for ampicillin in Figure 2, and chose the important 10-mers for all 15 antibiotics. Expanding these 10-mers to 20-mers, though not exhaustive, is computationally efficient and is also sufficient for achieving good prediction accuracy, thereby validating the approach.

**Figure 3:**
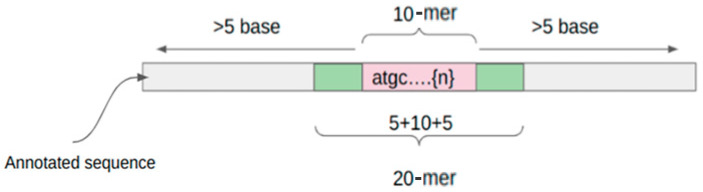
The 10-mer Expander searches for a 10-mer and extends both ends by 5 nucleotides to generate 20-mers.

While considering the identification of antibiotic responsive genes using k-mers, we know that shorter k-mers, such as those of length 10, may present challenges when attempting to perform BLAST searches for the identification of such resistant genes. Therefore, we made the decision to increase the k-mer length to enhance our ability to accurately identify antibiotic responsive genes. To achieve this, in the third step of the algorithm pipeline, we equally expanded each 10-mer on either side to obtain the 20-mer, as shown in Figure 3. We also experimented with all 11 extending options, but the improvement in MIC prediction accuracy was minimal from choosing the 10-mer in the center. This extension allowed us to significantly improve the specificity and sensitivity of our approach. As a result, when we expanded the 1352 ‘important’ 10-mers into 20-mers, we obtained 27,932 unique 20-mers from the database, which is a far smaller number than all possible (≈10^9^ in our case) 20-mers. This approach provides a more comprehensive representation of potential antibiotic resistance gene sequences, addressing the limitations of shorter k-mers for this specific purpose.

In this last (fourth) step, we repeat the dimensionality reduction by using feature importance, similar to the second step. The expanded 20-mer data were trained in an MLP regressor instead of the RF (additional details in Section 2.2), with the MIC values used as target labels. After training the model, we used DeepLift [27] to extract features (specific 20-mers and the genes that they aligned to; see more details of individual results in Section 3.1) that contribute to the MIC in a positive or negative manner. We have only listed the 10 most significant 20-mers (genes) for each of the antibiotics. We integrated traditional machine learning (RF) as well as a Multilayer Perceptron (MLP) sequentially to select and refine the important genomic features that affect the MIC prediction of *Salmonella* in this dataset. We are using the counts of the k-mers in our algorithm rather than just binary presence–absence indicators, as reported earlier [19].

### 2.2. RF and MLP

Random forest [28,29] is a powerful ensemble algorithm, which is a collection of individual decision trees that collaborate to improve the classification or regression task. Decision-tree-based algorithms are sensitive to the training data [30] and have low bias but a high variance [31]. In a random forest, this sensitivity is addressed by constructing multiple decision trees from random samples selected from the dataset, often with replacement. Predictions (regression) are then derived through a majority vote (average) from the ensemble of trees. In our analysis, we utilized 100 trees in the RF, using the Scikit-learn library (v 1.2.2).

The MLP network had 4 hidden layers, and each layer had 1024 neurons. Since the network’s aim is to obtain a regression on the MIC value, which is a positive variable with gaps in its range, (1) the final output layer had only one neuron with a linear activation function, while the other layers used a ReLU activation, and (2) the loss function used was MSE with an ADAM optimizer. For regularization, we used both batch normalization (BN) and dropout (DR). BN adjusts the values of units individually for each batch using their respective mean and standard deviations, while DR randomly deactivates a fraction (0.3) of units within the network. BN and DR help control scaling and overfitting, respectively. For both the RF and the neural network, we used an 80–20% train-test split and estimated test accuracy.

## 3. Results and Discussion

### 3.1. MIC Prediction Accuracy

For advancing personalized and effective antibiotic treatments, two notable limitations are (1) that the laboratory determination of MIC, crucial for tailored treatment, often takes a substantial amount of time, usually 3 to 5 days [32], and (2) that the MIC values exhibit natural variability, mainly due to genetic differences among pathogenic strains [33]. Genetic makeup significantly influences pathogen susceptibility to antibiotics.

In this study, we used integrated traditional machine learning (RF) and deep learning (multilayer perceptron—MLP) to predict *Salmonella* MIC values based on 20-mer counts in data from WGS. We reduced the dimension of a million 10-mers to produce 27,932 unique 20-mers, and we identified the top 10 20-mers that are predictive of the increase and decrease in MIC using our innovative Genome Feature Extractor Pipeline. The average prediction accuracy of MICs for 15 antibiotics over the entire 4500 genomes dataset is >96% (Table 2). The lowest accuracy (89.67) was obtained with sulfisoxazole, which had the largest range of MIC values, and that probably caused a large mean square error (MSE) in the predictor outputs. The MIC prediction for at least 10 of the 15 antibiotics was at ≥96.4% prediction accuracy and low MSE. These results underscore the importance of the dimension reduction and filtering of k-mers as critical steps in optimizing the performance of MIC prediction models.

Considering potential sequencing variations, it has been recommended to construct prediction models based on well-controlled experiments using WGS data sourced from the same laboratories [34]. The study achieved an average prediction accuracy of 92% for 24 antibiotics, with 321 WGS as predictors. In contrast, our approach, leveraging 4500 WGS, demonstrated an accuracy exceeding 96%. This suggests that variation in WGS originating from different labs could be less critical when there are ample data points, allowing machine learning models to learn more effectively. Furthermore, from a prediction perspective, lower variability in data can enhance accuracy but may compromise the model’s robustness by increasing sensitivity to variations. While WGS is effective for predicting AMR, the presence of a heteroresistant subpopulation in *Salmonella* enterica, exhibiting variability in sensitivity to an antimicrobial agent, could lead to an incorrect indication of an absence of resistance [35]. This introduces a notable limitation in machine learning, as the models might struggle to decipher the presence of heteroresistance when making MIC predictions. However, the results of our study align with previous predictive learning models that utilized 10-mer counts from the PATRIC database data. The earlier models, utilizing deep learning (neural network) [36] and traditional machine learning (XGBoost) [19], achieved prediction accuracies within the range of 85% to 95%. In our study, we employed both deep learning and random forest approaches for comprehensive analysis. Furthermore, in contrast to the XGBoost feature importance analysis that identified important k-mers for MIC prediction [19], our study employs the DeepLift technique to categorize the identified k-mers as specifically crucial for either high or low MIC values. Detailed discussions of these observations are included in the following sections.

### 3.2. Identification of Genomic Features Predictive of Antibiotic Susceptibility/Resistance

**Figure 4:**
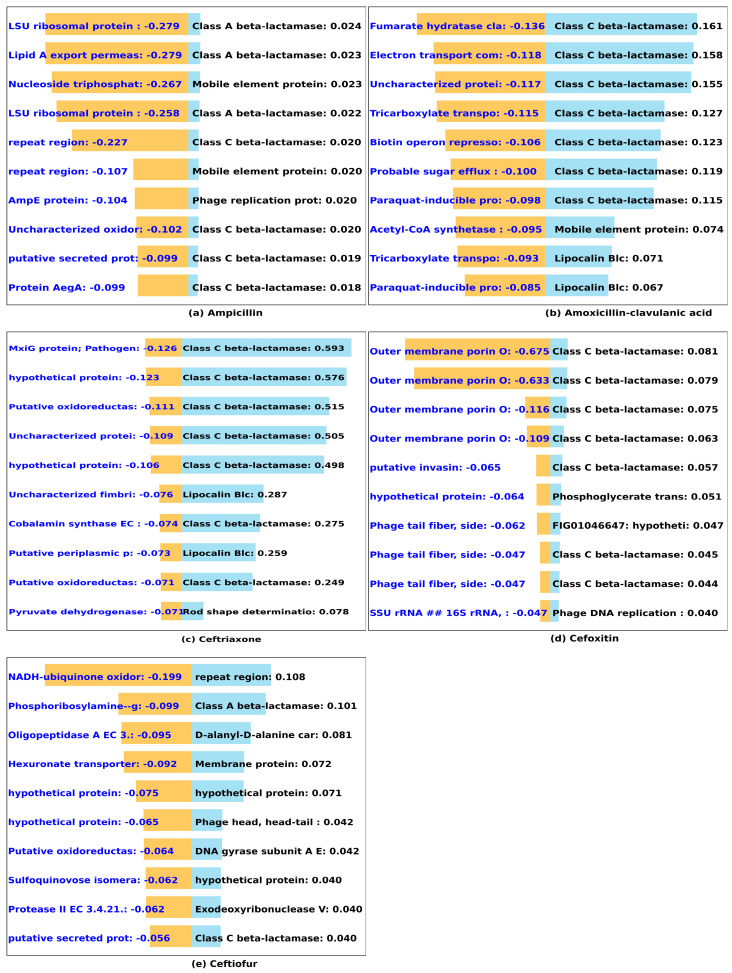
Predicted impact of k-mers on MIC of β-lactams antibiotics. We used DeepLift to predict whether k-mers (20-mers) increase (blue) or decrease (orange) MIC for (**a**) ampicillin, (**b**) amoxicillin-clavulanic acid, (**c**) ceftriaxone, (**d**) cefoxitin, and (**e**) ceftiofur. The bar graphs show the *Salmonella* genes that the kmers align to, and the length of the bar based on the importance score.

**Figure 5:**
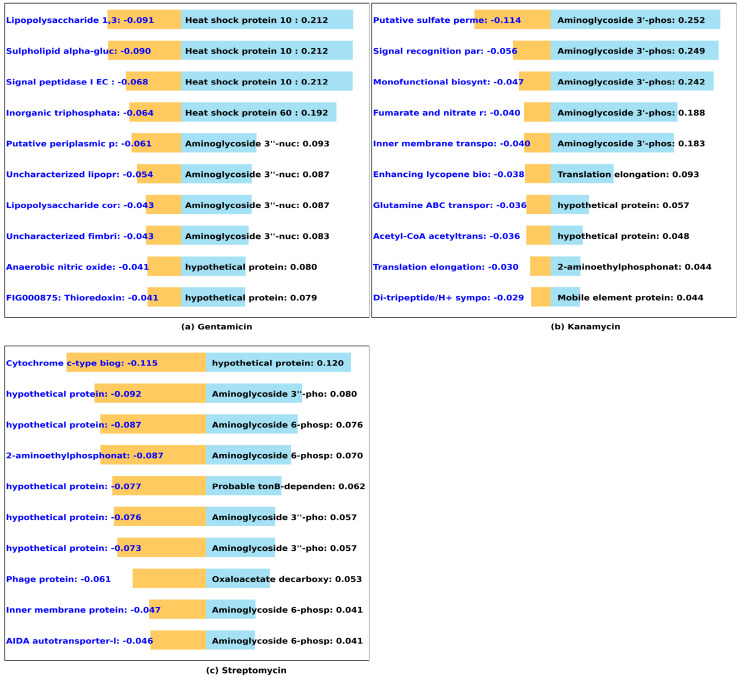
Predicted impact of k-mers on MIC of aminoglycoside antibiotics. We used DeepLift to predict whether k-mers (20-mers) increase (blue) or decrease (orange) MIC for (**a**) gentamicin, (**b**) kanamycin, and (**c**) streptomycin. The bar graphs show the *Salmonella* genes that the k-mers align to, and the length of the bar based on the importance score.

**Figure 6:**
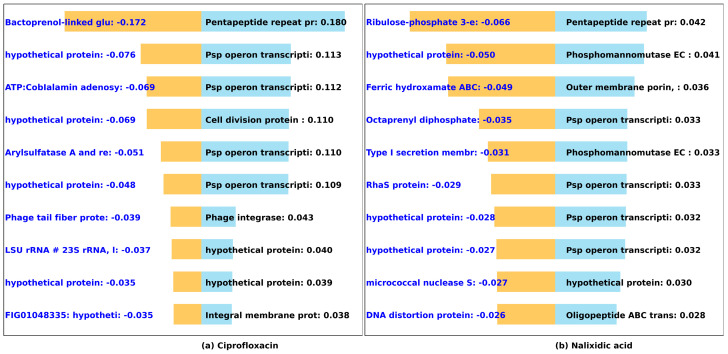
Predicted impact of k-mers on the MIC of quinolone antibiotics. We used DeepLift to predict whether k-mers (20-mers) increase (blue) or decrease (orange) MIC for (**a**) ciprofloxacin and (**b**) nalixidic acid. The bar graphs show the *Salmonella* genes that the k-mers align to, and the length of the bar based on the importance score.

**Figure 7:**
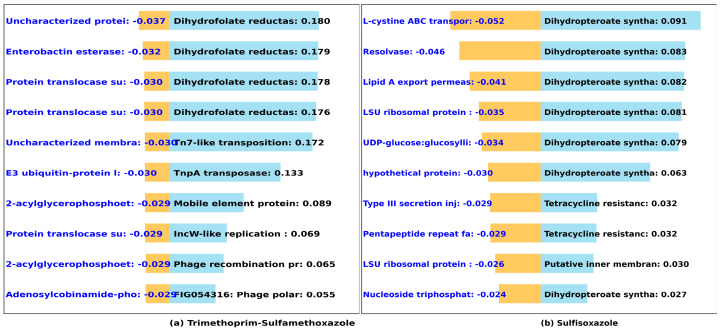
Predicted impact of k-mers on the MIC of sulfonamide antibiotics. We used DeepLift to predict whether k-mers (20-mers) increase (blue) or decrease (orange) MIC for (**a**) Trimethoprim-sulfamethoxazole and (**b**) sulfisoxazole. The bar graphs show the *Salmonella* genes that the k-mers align to, and the length of the bar based on the importance score.

**Figure 8:**
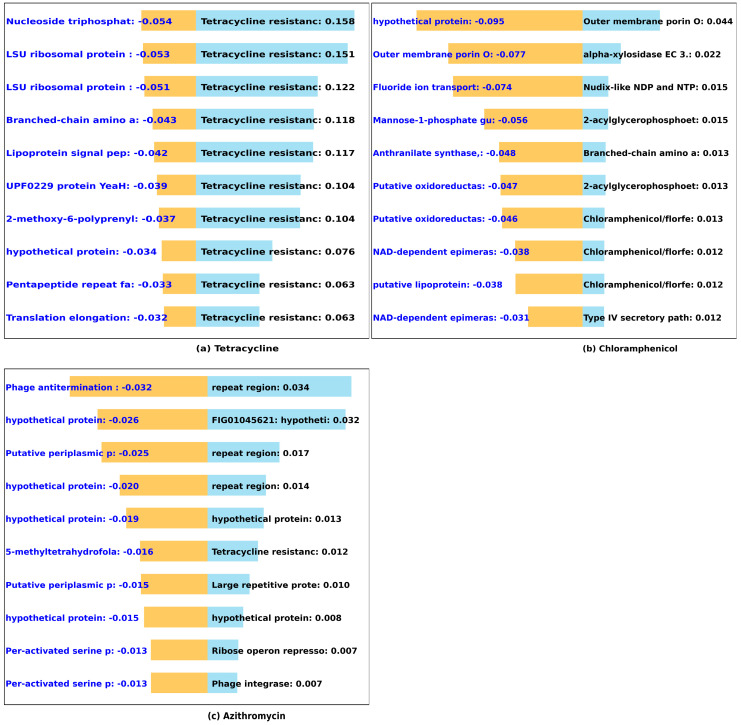
Predicted impact of k-mers on MIC of individual class of antibiotics. We used DeepLift to predict whether k-mers (20-mers) increase (blue) or decrease (orange) MIC for (**a**) tetracycline, (**b**) chloramphenicol, and (**c**) azithromycin. The bar graphs show the *Salmonella* genes that the k-mers align to, and the length of the bar based on the importance score.

A positive correlation between the presence of known antibiotic genes and laboratory-determined MIC values was shown in [37]. Furthermore, additional investigations have delved into the use of single nucleotide polymorphisms (SNPs) within known antibiotic genes to predict susceptibility and resistance [14,38,39]. However, these previous approaches often overlooked the potential contribution of novel genes or k-mers to MIC values. We hypothesized that the use of the frequencies (occurrence counts) of “important” k-mers to predict MIC values could generate novel gene/k-mer relevance to MIC values. Our analysis of the 15 antibiotics is categorized based on the set of known resistance genes to which they belong, see Table 1, with visual representation of the results, identified kmers and genes, in Figure 4, Figure 5, Figure 6, Figure 7 and Figure 8.

#### 3.2.1. β-Lactams (Ampicillin, Amoxicillin-Clavulanic Acid, Ceftriaxone, Cefoxitin, Ceftiofur)

We observed that seven, seven, seven, seven, and two out of ten crucial 20-mers are, respectively, associated with high ampicillin, amoxicillin-clavulanic acid, ceftriaxone, cefoxitin, and ceftiofur. MICs are prominently located within the Class A and C β-lactamases, as shown in Figure 4a–e. This finding is in alignment with the well-established association of Class A and C β-lactamases with penicillin resistance [40]. Our model identifies other genes encoding protein, such as mobile element protein and lipocalin that have been implicated in antibiotic resistance. While lipocalin has been computationally predicted to play an essential role in antibiotic resistance in *Salmonella*, as indicated by previous studies [19,41], investigation through both in vitro and in vivo analysis has confirmed that the presence of lipocalin extracted from *Burkholderia cenocepacia* can indeed induce resistance to quinolone and β-lactam antibiotics [42]. Mobile elements, often called mobile genetic elements, are segments of DNA that can move around in a genome. They can carry genes, including antibiotic resistance genes, and facilitate their spread among bacteria. The capability of these mobile elements to transport resistance genes within *Salmonella* has been well-established [43]. While the direct roles of other important identified k-mers/genes in β-lactam antibiotic resistance may not be evident, it is worth noting that d-alanyl-d-alanine carboxypeptidase is known to be involved in cell wall synthesis in *Streptomyces coelicolo* [44]. Furthermore, exonuclease activity associated with DNA repair in *Salmonella* [45] may contribute to its overall fitness and ability to withstand β-lactam antibiotics. Upon examining the genes predicted to contribute to low values of β-lactam MIC, indicative of susceptibility, our model identified AmpE, a well-known negative regulator of β-lactamase in *E. coli* [46] and *Pseudomonas aeruginosa* [47]. These findings underscore the robustness and versatility of our approach in antibiotic susceptibility and/or resistance prediction.

#### 3.2.2. Aminoglycosides (Gentamycin, Kanamycin, Streptomycin)

Aminoglycoside phosphotransferases and nucleotidyltransferase [48,49,50] are well-established resistance genes emerging as the top predictors, seven, five, and four out of ten, respectively, for high MIC values in streptomycin, kanamycin, and gentamicin, as shown in Figure 5. This alignment between our predictions and established knowledge underscores the reliability of our model in capturing essential antibiotic resistance mechanisms. While the MIC dependence on heat shock proteins family genes may not be direct, they have been shown to be involved in protein folding and stability, as well as stress response [51,52] and biofilm formation [53]. which could ultimately impact antibiotic resistance.

#### 3.2.3. Quinolones (Ciprofloxacin, Nalixidic Acid)

Using RF, mutations in DNA gyrase genes *gyrA*, *parC*, and quinolone resistance gene *qnrS* have been identified as predictors of quinolone resistance in *E. coli* [54]. The plasmid-mediated quinolone resistance gene B (*qnrB*) encodes proteins belonging to the pentapeptide repeat family gene [55]. These proteins safeguard DNA gyrase and topoisomerase IV against inhibition by quinolone antibiotics. The pentapeptide protein, associated with the high MIC values for ciprofloxacin and nalixidic acid, as illustrated in Figure 6, is a well-established quinolone resistance determinant [56,57,58]. Furthermore, we observed that the presence of the phage shock protein (PSP) operon, necessary to maintain membrane integrity, contributes to high MIC values of quinolone antibiotics in this study. The upregulation of PSP has been linked to quinolone resistance in *E. coli* in [59]. In addition, significant upregulation of outer membrane protein genes is associated with resistance to quinolones in *Salmonella Typhi* [60]. This study identifies outer membrane porin, a type of outer membrane protein, as important for nalixidic (quinolone) resistance (Figure 6).

#### 3.2.4. Sulfonamides (Trimethoprim-Sulfamethoxazole, Sulfisoxazole)

Dihydrofolate reductase (four out of ten important genes) and dihydropteroate synthase type-2 (seven out of ten of the important genes) (Figure 7) are the principal contributors to high MIC in trimethoprim-sulfamethoxazole and sulfisoxazole, respectively [61]. These genes are well known to confer resistance to sulfonamides in *Salmonella* genomes [62]. Mutations in both dihydrofolate reductase and dihydropteroate synthetase have been demonstrated to elevate *Plasmodium falciparum* resistance to sulfadoxine-pyrimethamine, a known sulfonamide antibiotic [61]. Furthermore, we observed that tetracycline resistance genes, and transposase, linked to antibiotic resistance [63,64] appear to play a significant secondary role in *Salmonella* resistance to sulfisoxazole and trimethoprim-sulfamethoxazole in our dataset.

#### 3.2.5. Individual Antibiotic Class (Tetracycline, Chloramphenicol, Azithromycin)

Tetracycline, chloramphenicol, and azithromycin belong to the tetracycline, chloramphenicol, and macrolide class, respectively. In the case of tetracycline (Tet), all identified genes indeed encode essential components, including major facilitator superfamily (MFS) efflux Tet (A) and Tet (B) resistance genes, as well as the tetracycline regulatory gene involved in tetracycline resistance (Figure 8). Similarly for chloramphenicol, our model identity the presence of chloramphenicol resistant genes, as expected. However, despite the success of our model in identifying several established resistance genes in 14 different antibiotics across six classes, it could only identify one resistance gene belonging to tetracycline class in our azithromycin (macrolide) model. This may not be totally surprising as macrolide resistance genes such as erythromycin ribosome methylation (erm) gene are often carried on the extrachromosomal plasmid [65,66].

In summary, we introduce the “Genome Feature Extractor Pipeline” as a novel solution to the challenges posed by employing 10-mers for minimum inhibitory concentration (MIC) prediction. While 10-mers serve well for predicting MIC values, their utility diminishes when used in BLAST searches for pinpointing genomic regions influencing MIC values. Our innovative pipeline addresses this issue by effectively reducing the dimensionality of the massive pool of approximately one million 10-mers. It does so by transitioning these 10-mers into a more manageable set of tens of thousands of 20-mers, specifically tailored to encapsulate the genomic regions that exert a significant influence on MIC values. This approach not only helps us to navigate the computational complexities associated with working with thousands of genomes, but also unveils a clear understanding of the genomic features that drive antibiotic susceptibility and resistance. Moreover, our tool exhibits a remarkable ability to discriminate the specific contributions of essential 20-mers and the genes in which they are embedded. This level of discrimination is instrumental in elucidating the roles these genetic elements play in determining low MIC values, indicative of susceptibility, or high MIC values, indicative of resistance, within a dataset comprising 4500 genomes. Importantly, many of the k-mers and genes predictive of resistance to β-lactam, aminoglycosides, quinolones, sulfonamides, chloramphenicols, and tetracyclines, as identified by our learning model using a combination of random forest, multilayer perceptron, and DeepLift techniques, are consistent with known resistance mechanisms reported in the scientific literature. Finally, our model extends its contribution to the identification of genes encoding various proteins, including lipocalin, heat shock protein, mobile elements, phage shock protein, and several hypothetical proteins. These proteins hold the potential to play a role in conferring antibiotic resistance in *Salmonella*. However, their actual contribution needs validation through rigorous experimental studies, a scope that extends beyond the focus of this study.

## Figures and Tables

**Table 1:** Antibiotics and their known biological target, and the associated group of resistance genes.

Antibiotic	Target	Resistance Genes Group
Ampicillin	Cell Wall	β-lactam
Amoxicillin-clavulanic acid	Cell Wall	β-lactam
Ceftriaxone	Cell Wall	β-lactam
Azithromycin	Protein	Macrolide
Chloramphenicol	Protein	Phenicol
Ciprofloxacin	DNA	Quinolone
Trimethoprim-Sulfamethoxazole	DNA	Sulfonamide
Sulfisoxazole	DNA	Sulfonamide
Cefoxitin	Cell Wall	β-lactam
Gentamicin	Protein	Aminoglycoside
Kanamycin	Protein	Aminoglycoside
Nalixidic acid	DNA	Quinolone
Streptomycin	Protein	Aminoglycoside
Tetracycline	Protein	Tetracycline
Ceftiofur	Cell Wall	β-lactam

**Table 2:** Multilayer perceptron prediction accuracy of MIC for 15 antibiotics.

	Antibiotic	Prediction Accuracy	MSE for Prediction	MIC Range
1	Ampicillin	96.89	0.54	1–32
2	Amoxicillin-clavulanic acid	97.44	0.35	1–32
3	Ceftriaxone	97.46	0.17	0.25–64
4	Azithromycin	96.37	0.28	1–16
5	Chloramphenicol	97.44	0.21	2–32
6	Ciprofloxacin	98.20	0.16	0.01–2
7	Trimethoprim-Sulfamethoxazole	98.56	0.16	0.12–4
8	Sulfisoxazole	89.67	0.48	16–2048
9	Cefoxitin	93.00	0.33	1–32
10	Gentamicin	92.33	0.64	0.25–16
11	Kanamycin	97.5	0.23	8–64
12	Nalixidic acid	95.78	0.27	1–64
13	Streptomycin	94.14	0.47	2–64
14	Tetracycline	99.11	0.21	4–32
15	Ceftiofur	96.67	0.18	0.25–8

## Data Availability

Publicly available datasets from PATRIC database (now known as Bacterial and Viral Bioinformatics Resource Center) were analyzed in this study. These data can be found here: https://www.bv-brc.org/ (accessed on 15 October 2023).
